# Antifungal Effect of Polymethyl Methacrylate Resin Base with Embedded Au Nanoparticles

**DOI:** 10.3390/nano13142128

**Published:** 2023-07-22

**Authors:** Ivan Marić, Anamarija Zore, Franc Rojko, Andrijana Sever Škapin, Roman Štukelj, Aleksander Učakar, Rajko Vidrih, Valentina Veselinović, Marijan Gotić, Klemen Bohinc

**Affiliations:** 1 Faculty of Health Sciences, University of Ljubljana, 1000 Ljubljana, Slovenia; imaric@irb.hr (I.M.); anamarija.zore@zf.uni-lj.si (A.Z.); franc.rojko@zf.uni-lj.si (F.R.); roman.stukelj@zf.uni-lj.si (R.Š.); 2 Ruđer Bošković Institute, 10000 Zagreb, Croatia; marijan.gotic@irb.hr; 3 Slovenian National Building and Civil Engineering Institute, 1000 Ljubljana, Slovenia; andrijana.skapin@zag.si; 4 Faculty of Polymer Technology-FTPO, Ozare 19, 2380 Slovenj Gradec, Slovenia; 5 Institute Jožef Stefan, Jamova Cesta 39, 1000 Ljubljana, Slovenia; aleksander.ucakar@gmail.com; 6 Biotechnical Faculty, University of Ljubljana, 1000 Ljubljana, Slovenia; rajko.vidrih@bf.uni-lj.si; 7 Faculty of Medicine, University of Banja Luka, 78000 Banja Luka, Bosnia and Herzegovina; valentina.veselinovic@med.unibl.org

**Keywords:** fungal adhesion, *C. albicans*, polymethyl methacrylate resin, Au, surface properties, composites

## Abstract

Full and partial restorations in dentistry must replicate the characteristics of the patient’s natural teeth. Materials must have good mechanical properties and be non-toxic and biocompatible. Microbes, which can form biofilms, are constantly in contact with restorations. In this study, we investigate how well *Candida albicans* adheres to a polymethyl methacrylate (PMMA) resin base with gold (Au) nanoparticles. We synthesized Au nanoparticles and characterized them. The average size of Au nanoparticles embedded in PMMA was 11 nm. The color difference ΔE between PMMA and PMMA/Au composites was 2.7 and was still esthetically acceptable to patients. PMMA/Au surfaces are smoother and more hydrophilic than pure PMMA surfaces, and the isoelectric point of both types of surfaces was 4.3. Above the isoelectric point, PMMA/Au surfaces are more negatively charged than PMMA surfaces. The added Au nanoparticles decreased the tensile strength, while the hardness did not change significantly. Adhesion measurements showed that PMMA surfaces modified with Au nanoparticles reduced the extent of microbial adhesion of *Candida albicans*.

## 1. Introduction

The number of old people in the total world population is increasing rapidly and, according to United Nations data, it will reach as much as 16% by 2050 [1]. The rapidly growing number of old people that are partially or completely toothless, who are wearers of acrylate mobile prostheses, can partly explain the very high prevalence of prosthetic stomatitis, as much as 67% [2,3,4]. Inflammation and erythema of the supporting denture tissues that the prosthesis lay on are the main symptoms of this disease. Despite the high prevalence, the etiology of prosthetic stomatitis has not been fully elucidated [2,5]. The mechanism of development of denture stomatitis is multifaceted and it is based on the interaction between predisposing local and systemic factors [5]. Local factors favoring the development of denture stomatitis are mouth dryness, local trauma caused by inadequate denture fitting, poor oral hygiene, continuous denture wearing, low saliva pH [6], and smoking [7]. Research has shown that among the mentioned factors, poor oral hygiene and continuous denture wearing represent the most significant local risk factors [8]. It is known that mobile acrylate prostheses covering the supporting soft tissues reduce the flow of oxygen and saliva to the underlying tissue, creating suitable local conditions for the growth of *Candida albicans* colonies [9]. Systemic predisposing factors, which increase individual susceptibility to fungal infection, include poor nutrition, immunosuppression, and hematological diseases [6].

Polymethyl methacrylate, thanks to its positive properties such as high biocompatibility, low density and weight, satisfactory aesthetics with the possibility of matching the color of natural tissues that compensate, satisfactory mechanical and physical properties, simple technology of use, the possibility of polishing, and reasonable price, represents the material of choice for dental denture base productions [10]. PMMA-based materials are aesthetically pleasing, inexpensive, simple to shape and manipulate, and they have good biocompatibility and stability in the oral cavity making them especially important in the field of dentistry [10]. In contrast to its advantages, PMMA suffers from limitations such as poor antimicrobial properties, high water absorption, low conductivity, and limited resistance to mechanical fatigue. These limitations are topics of numerous scientific studies [11,12,13,14]. However, the most important drawback of denture base polymethyl methacrylate, viewed from the health aspect, is its susceptibility to the accumulation of microbial biofilms [15].

*Candida albicans* is the most common fungal pathogen in the human population and the main cause of denture stomatitis. As a commensal microorganism, it can be found in the oral cavity of 45–65% of people who have no health issues. Studies have shown that the prevalence of *Candida albicans* increases up to 60–100% in people who wear dentures [5]. *Candida albicans* cells are predominantly organized into a biofilm structure with high cell density, adhered to tissues. The biofilm structure is very often resistant to the action of even high doses of antifungal drugs. Drug resistance is determined by multiple mechanisms [16] and its specific extracellular matrix that protects it is cited as one of the main factors [17]. 

Microorganisms that release extracellular vesicles that deliver RNA and proteins protected by the surrounding lipid bilayer are responsible for the production of the extracellular matrix. Extracellular vesicles differ based on size, materials they carry, and mechanisms of biogenesis [18,19]. The biological role of extracellular vesicles depends on the target cell, and it is believed that through them the microorganism cell can, for example, deliver a toxin to the target cell and thus cause its lysis and death [18]. Extracellular biofilm vesicles are essential for the formation of the matrix and the development of drug resistance according to a study by Zarnowski et al. [20].

Although prosthetic stomatitis is a relatively non-dangerous disease that cannot cause more serious complications in a healthy population, we must consider the fact that patients with developed prosthetic stomatitis are mostly older people, with poorer general health and multiple comorbidities, which put prosthetic stomatitis into the order of potentially dangerous diseases [21]. More than a million people are thought to die each year from fungal infections, mostly those brought on by species of *Candida, Cryptococcus,* and *Aspergillus*. Despite the existence of numerous antifungal therapies, these overlooked diseases are difficult to treat and have a very high death rate [22]. Given that the number of denture wearers on a global level is very high, with almost 41 million users in the United States alone in 2020 [23], an accelerated effort by the world research community is necessary to solve the problem of *Candida albicans* infection and denture stomatitis [24]. 

Microbial adhesion is governed by both microbial properties and the properties of the dental material surface. Therefore, it is important to design materials with optimized properties (hydrophobicity, charge, surface roughness, etc.) to inhibit microbial adhesion. Negatively charged surfaces tend to be more resistant to microbial adhesion due to the negative charge of microbial cells. It was noticed that less biofilm forms on hydrophobic supragingival surfaces, while no difference was noticed between hydrophobic and hydrophilic subgingival surfaces [25]. Surface roughness is deemed a crucial factor in microbial adhesion. Increasing surface roughness provides microbial cells better contact with the surface and easier attachment, while smoothening the surface reduces their ability to attach to the surface and form a biofilm [26,27]. 

Bohinc et al. showed that between five stainless steel surfaces with varying surface roughness, the highest degree of bacterial adhesion was on the rougher surfaces [28]. Conventional drugs used in prosthetic stomatitis therapy caused by *Candida albicans*, such as nystatin, amphotericin B, ketoconazole, miconazole, fluconazole, itraconazole, clotrimazole, and others, show efficacy in a short period of time but cause toxic side effects, formation of resistant strains [29], and the infection soon recurs. Inadequate and excessive use of antibiotics and antifungal drugs in all fields of medicine and dentistry has led to the drug resistance of target microorganisms as one of the biggest problems with which modern medicine is struggling today [30]. In addition, the constant flow of saliva washes away the therapeutic agent and reduces its effect. There is a need for the rapid development of new antifungal drugs because the currently available ones are limited in their effectiveness and do not provide safe and long-lasting protection. It is necessary to take advantage of the current rich knowledge about the fungal cell’s physiology and the mechanisms of infection and use the knowledge resources for the development of new generations of antifungal drugs [29].

One of the most current and promising directions in the development of new antimicrobial strategies is the development of denture base materials that will have their own antimicrobial activity [31]. The rapid development of nanotechnology and nanomaterials has enabled the modification of conventional polymethyl methacrylate by adding different forms of nanostructural elements such as fibers, nanoparticles, and other forms of nanofillers [32]. Metallic nanoparticles [33], metal oxide nanoparticles [34], and carbon-based nanoparticles [35] represent the most often used nanoparticles for the modification of PMMA materials in order to obtain composites of optimal biological, optical, and mechanical properties [10,13]. Zore et al. [13] have shown that TiO_2_ fillers reduce the bacterial coverage of PMMA/TiO_2_ composites, however, the tensile strength decreased with the increase of the TiO_2_ fillers up to 20%. Silver nanoparticles have also been used as an effective filler and their antimicrobial properties are well described in the literature [36,37]. Akhavan et al. developed PMMA/Ag composites and showed that these materials completely inhibit the growth of *E. coli* unlike pure PMMA where bacterial growth readily occurs [38].

On the other hand, Au NPs have not been extensively explored for this application [11]. Bearing in mind the ancient, traditional use of gold for therapeutic purposes and its biocompatibility, the development of nanotechnology and the possibility of synthesizing gold nanoparticles with a large reactive surface has opened new, very promising applications of gold [39]. Gold nanoparticles can be routinely synthesized by various methods such as the classical citrate method [40], UV irradiation [41], ultrasonic spray pyrolysis [42,43], and γ-irradiation [44]. Over the years, the classical citrate method was refined, and it became possible to synthesize gold nanoparticles with well-controlled size and monodispersity. For example, Schulz et al. have shown that optimization of sodium citrate/citric acid buffer ratio yielded reproducible NPs between multiple batches [45]. One of the most fascinating characteristics of AuNPs are their optical properties which are dependent on their size, shape, and surface chemistry. Therefore, the color of AuNPs can be finely tuned to match the color of human target tissue, as this is very important from an aesthetic point of view [46]. Furthermore, Au NPs can improve the thermal conductivity of composite materials [11,47]. Other key advantages of Au NPs are their antimicrobial properties. Sawant et al. showed that incorporation of Au NPs into different polymeric materials reduced the growth of *E. coli* by a factor of 10^6^ compared to unmodified surfaces [48]. Several authors also noted a pronounced antifungal property of Au NPs on different *Candida* species, such as *C. albicans, C. glabrata*, and *C. tropicalis* [49,50,51]. 

Generally, work relating to the antifungal properties of PMMA/Au medical materials is scarce. A notable example is the work of Russo et al. who showed that the inclusion of Au NPs into PMMA-based bone cement up to 1% *w/w* almost completely inhibited the growth of a bacterial biofilm [52].

Therefore, in this work, we performed a complete and extensive investigation of the possibility of using Au NPs as modifiers of PMMA dental materials and explored their antifungal properties by means of *C. albicans* adhesion experiments. In this work, Au NPs were synthesized by the simple and inexpensive citrate method, keeping in mind the necessity to reduce the manufacturing cost of such materials and to keep them accessible to a wider population. Streaming potential, hydrophobicity, and quantitative color measurements were evaluated. All studied parameters are important to consider when designing novel materials for dental uses, although are often neglected.

## 2. Materials and Methods

### 2.1. Materials

All chemicals were of analytical purity and used as received. Gold(III) chloride trihydrate (≥99.9% trace metals basis), citric acid (puriss., Reag. Ph. Eur.), and trisodium citrate dihydrate (for analysis EMSURE, ACS, ISO, Reag. Ph. Eur.) were provided by Sigma-Aldrich. Methyl methacrylate monomer (Ivoclar, Schaan, Liechtenstein), PMMA (Ivoclar, Schaan, Liechtenstein) were also used. Milli-Q deionized water was used for all experiments.

#### 2.1.1. Preparation of Au Nanoparticles

The Au NPs were synthesized according to a previous procedure [45] described by Schulz et al. In short, 150 mL of a sodium citrate/citric acid buffer was prepared in a ratio of 75/25 with 2.2 mM being the total concentration of citrates. This solution was heated to reflux and boiled for 15 min. A total of 1.225 mL HAuCl_4_ precursor solution (20.4 mM) was quickly added to the boiling solution while it was continuously and vigorously stirred. A change in color immediately occurred, forming a wine-red solution within minutes. At this point, the heating mantle was turned off, but the flask was left on the heating mantle for another 15 min, after which the flask was removed from the heating mantle and placed in a cold-water bath. After cooling, the suspension was kept in the dark and refrigerated at 4 °C.

#### 2.1.2. Preparation of PMMA and PMMA/Au Films

For the fabrication of Au-modified PMMA, a 20% Au suspension and 80% monomer in a total volume of 3 mL was mixed with 6 g of polymer in the powder form. After, the same procedure as above was used. For mechanical testing, 15 mm × 80 mm pieces with thickness of 3 mm were prepared. For all other analyses, 10 mm × 10 mm pieces with thickness of 3 mm were prepared.

#### 2.1.3. Fungi/Yeast

A clinical strain of *C. albicans,* obtained from the Institute of Microbiology and Immunology, University of Ljubljana, was used. The strain was selected from a culture grown on Yeast Glucose Chloramphenicol Agar (YGC) (Merck KGaA, Darmstadt, Germany), and incubated at 37 °C for 48 h. An overnight culture of *C. albicans* was prepared in a Sabouraud nutrient broth (Biolife Italiana, Milano, Italia) at 37 °C for 19 h to achieve a yeast suspension concentration of 10^9^ CFU/mL. 

### 2.2. Methods

#### 2.2.1. UV-Vis Spectroscopy

The colloidal solution of Au NPs was analyzed using a Cary Varian 50 Bio spectrophotometer. The spectrum was collected in a wavelength range of 800 to 200 nm. The average particle size and colloid concentration were estimated according to a paper by Haiss et al. [53]. 

#### 2.2.2. CIE Colorimetry

Using a Minolta CR-400 colorimeter (Kyoto, Japan) and the L*a*b* three-dimensional color space of PMMA resins was assessed. Measurements of restorative materials were carried out in triplicates. To evaluate color differences, (ΔE) was calculated according to the equation ΔE = ([ΔE·a*]^2^ + [ΔE·b*]^2^ + [ΔE·L*]^2^)^1/2^, with ΔE classified as ‘very distinct’ for ΔE > 3, ‘distinct’ for 1.5 < ΔE < 3, and ‘nondistinct’ for ΔE < 1.5.

#### 2.2.3. Contact Angle Measurements

A Theta Optical Tensiometer (Attension, Finland) was used to measure the contact angle of the materials by the sessile drop method. The measurements were performed at 3.3 FPS in a 10 s window. The measurements were performed for 10 repetitions. The instrument was calibrated using a 4 mm diameter ball. 

#### 2.2.4. Zeta Potential Measurements

Using an electrokinetic analyzer (SurPASS, Anton Paar GmbH, Graz, Austria), the zeta potential of the produced films (2 × 1 cm) was determined as a function of pH at ambient temperature. To prevent CO_2_ from dissolving in the electrolyte solution during preparation and measurement, N_2_ was continuously bubbled into the solution. The pH of the electrolyte solution was adjusted using diluted HCl (0.05 M). The Helmholtz–Smoluchowski equation was used to calculate the zeta potential from the streaming current.

#### 2.2.5. Surface Morphology and Topography

The surface topography of PMMA/Au composite surfaces was measured by profilometry using a tribo profilometer from Tribotechnic, Clichy, France. The stylus was moved in a length of 4.8 mm with a scanning speed of 500 µm s^−1^. The measurements were repeated 5 times at different regions of the samples. A 0.8 mm Gaussian filter was applied to separate the waviness from the roughness. DIN EN ISO 4288:1998 standard was used.

#### 2.2.6. Mechanical Properties

The tensile properties of samples were measured according to EN ISO 527-3:2018. The Z030 apparatus (Zwick, Ulm, Germany) was used to perform uniaxial tensile testing on the materials that had been selected. The relative humidity and the temperature during testing were 50% and 23 °C, respectively. A 2 mm/min testing speed was used. Four measurements were taken for each type of sample. The test specimens had been conditioned for three days at the same temperature and relative humidity before the measurements. A preloading of 10 N was applied. The initial gauge length and the grips’ starting distance were separated by 40 mm. To measure the tensile strength of PMMA/Au composites, test pieces of PMMA/Au in the dimension of approximately 80 mm × 15 mm × 2.5 mm for each type were modeled. 

A shore D durometer (Zwick, Ulm, Germany) was used to determine the material’s hardness. On a scale from 0 to 100, the measured values represent the tested material’s resistance to indentation. On four separate films of each sample, a total of five measurements were made.

#### 2.2.7. Cell Adhesion

The overnight culture of *C. albicans* was diluted in a ratio of 1:30 in fresh Sabouraud nutrient broth to obtain an approximate concentration of ~10^7^ CFU/mL. Samples of pure PMMA and PMMA/Au composites were incubated with a cell concentration of ~10^7^ CFU/mL in 5 mL of yeast suspension for 24 and 48 h at 37 °C, respectively. After the designated incubation periods, the samples with attached yeast were rinsed 3 times in PBS buffer and 3 times in deionized water, and then fixed using hot air. 

#### 2.2.8. SEM Analysis and EDS Analysis

A scanning electron microscope (SEM, Quanta 650, ThermoFisher Scientific, Waltham, MA, USA) was used to analyze samples with adhered and fixed yeast. The samples were coated with a gold layer of approximately 5 nm thickness (BAL-TEC SCD 005, BALTEC AG, Pfäffikon, Switzerland). The gold coating process was performed at a vacuum of 5 × 10^−2^. The secondary electron sensor was used for imaging at a working distance of 10 mm at an accelerating voltage of 10 kV and vacuum of 2 × 10^−4^.

EDS elemental analysis of PMMA/Au samples (Ultim Max SDD 40 mm^2^, Oxford Instruments, Abingdon, UK) was made at 30 kV and WD = 10 mm at low magnification.

## 3. Results

### 3.1. UV-Vis Spectroscopy

**Figure 1:**
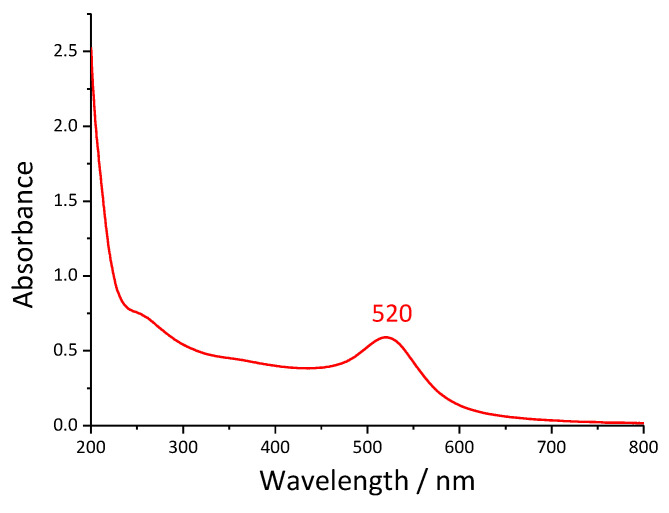
UV-Vis spectrum of the synthesized Au NPs.

The UV-Vis spectrum of the Au nanoparticle suspension is presented in Figure 1. The spectrum is characterized by a characteristic surface plasmon resonance peak (λ_SPR_) at 520 nm and a peak at ~250 nm. The peak at ~250 nm can be attributed to oxidation/degradation products of dicarboxyacetone [54], which is an important intermediate product and reducing agent in the classic Turkevich method [40,45]. 

### 3.2. CIE Colorimetry

**Figure 2:**
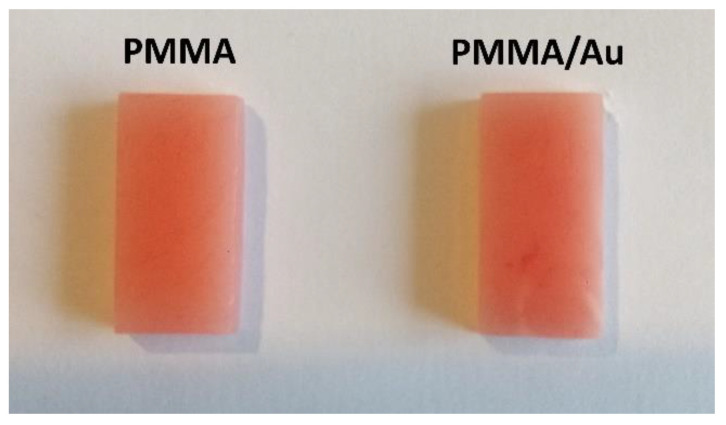
Photographs of PMMA (**left**) and PMMA/Au (**right**) composites.

The photographs of the two films can be seen in Figure 2. At a glance, there is no significant difference in color between the PMMA and PMMA/Au films, except in the uniformity of color at particular positions of the PMMA/Au sample. CIE colorimetry was performed to examine the color difference quantitatively. The L*, a*, and b* parameters of the CIE color space are presented in Table 1. As seen from these results, the addition of Au nanoparticles to PMMA increased the lightness, decreased parameter a* (less intensive red color), and decreased parameter b* (less intensive yellow color). 

### 3.3. Contact Angle Measurement Results

**Figure 3:**
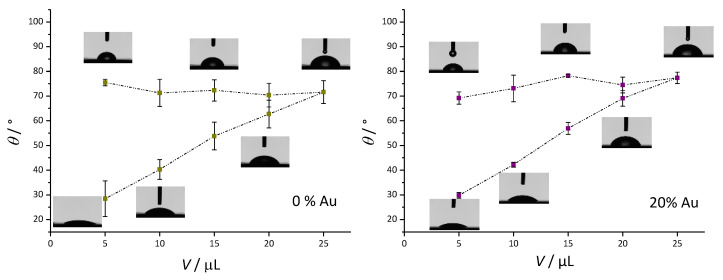
Advancing (θ_adv_) and receding (θ_rec_) water contact angles (θ_w_) as a function of water droplet volume (V_water_) for pure PMMA-based material (**left panel**) and composite PMMA/Au material (**right panel**) with accompanying photographs of the water droplets.

Figure 3 presents the dynamic contact angle measurements. For pure PMMA, the advancing contact angle changed from θ_(5 µL)_ = 75.52° ± 1.34° to θ_(25 µL)_ = 71.60° ± 4.57°. The receding contact angle decreased as the volume of the droplet decreased up to a final point of θ_(rec)_ = 28.36° ± 7.24°. The difference between the static contact angle and the final contact angle (contact angle hysteresis) was Δθ_w_ = 47.16°. For the PMMA/Au composite, the advancing contact angle increased slightly from θ_(5 µL)_ = 69.18° ± 2.53° to θ_(25 µL)_ = 77.43° ± 2.33°. The final receding contact angle was quite similar to the pure PMMA sample, θ_(rec)_ = 29.77° ± 1.18°, therefore, the contact angle hysteresis was Δθ_w_ = 39.41°, which was somewhat smaller compared to the pure PMMA sample. The static contact angle values showed that the addition of Au nanoparticles to PMMA slightly increased the hydrophilicity of the surface, whilst the relatively high contact angle hysteresis indicated that both samples had good wettability properties [55].

### 3.4. Streaming Potential Results

**Figure 4:**
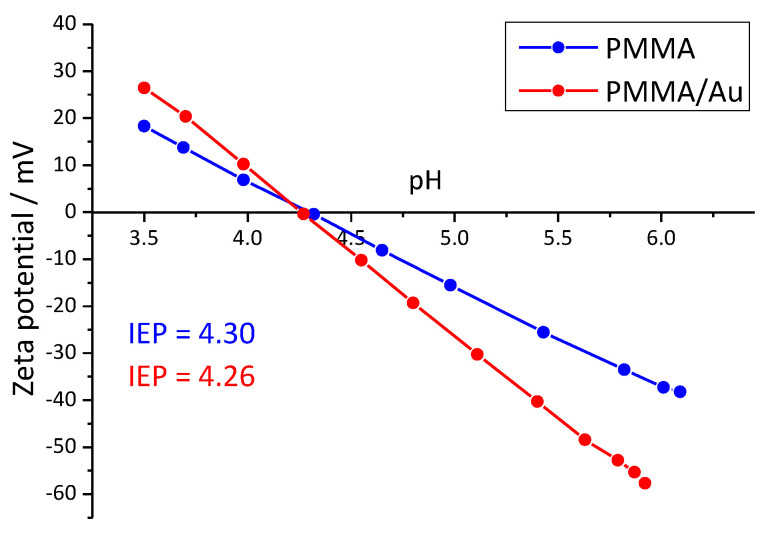
Zeta potential of PMMA (blue) and PMMA/Au (red) films as a function of pH.

The measured zeta potentials as a function of pH are shown in Figure 4. For both samples, the isoelectric point was at around pH = 4.30. Above the IEP, the surfaces were negatively charged, whereas the surfaces were positively charged below the IEP, as expected. However, PMMA/Au composites had higher magnitudes of zeta potential at the low and high end of the pH range. For example, at pH ~ 5.4, the zeta potential of PMMA/Au was −30.26 mV, whereas the zeta potential of pure PMMA was −25.55 mV.

### 3.5. Surface Roughness

**Figure 5:**
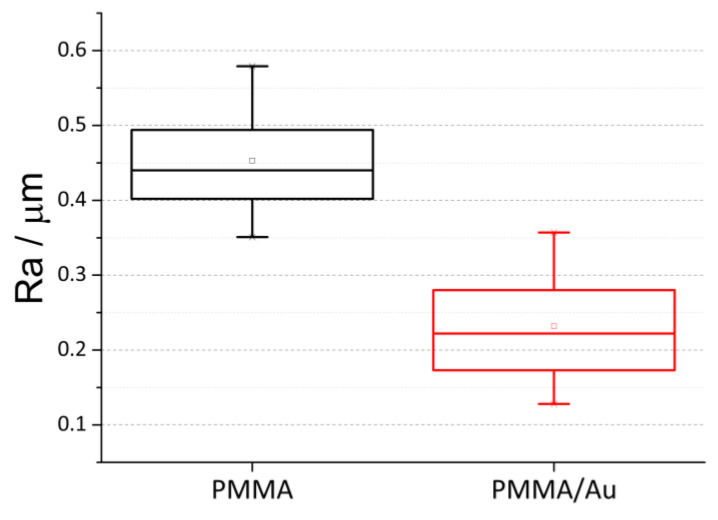
Surface roughness of PMMA and PMMA/Au samples.

A mechanical profilometer was used to analyze the roughness of both PMMA and the Au-modified PMMA material. Figure 5 shows the box-plot of five measurements for both samples. The roughness of the pure PMMA (Ra = 0.45 μm ± 0.09 μm) was significantly higher than the surface roughness of the PMMA/Au sample (Ra = 0.23 μm ± 0.09 μm).

### 3.6. Mechanical Properties

The mean values and standard deviations of measurements of the maximum tensile stress (σ_M_) and the corresponding strain (ε_M_), as well as the Shore D hardness values obtained by the Shore method are shown in Table 2. σ_M_ and ε_M_ were found at the end of the curves, at the break that occurred, for all measurements. PMMA without Au NPs had a value of σ_M_ (56.67 MPa) at ε_M_ of 3.87%. Upon the addition of 20% Au, the value of σ_M_ reduced to 34.73 MPa at ε_M_ of 2.78%. Interestingly, the hardness of the materials obtained by the Shore method expressed in the Shore D hardness scale did not change significantly; the Shore D value for pure PMMA was 22.2, while the Shore D value for PMMA/Au was 22.7.

### 3.7. Evaluation of Adhesion Extent of C. albicans

**Figure 6:**
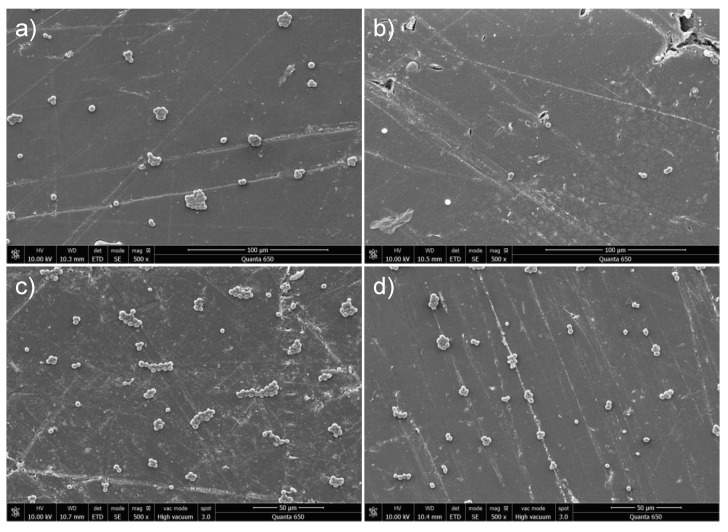
Representative SEM micrographs of *C. albicans* yeast after incubation of PMMA (**a**,**c**) and PMMA/Au (**b**,**d**) samples for 24 h (**a**,**b**) and 48 h (**c**,**d**).

The extent of yeast adhesion to the surfaces was assessed using SEM microscopy. Micrographs of material surfaces with attached fungi are shown in Figure 6. The images represent the extent of yeast adhesion after 24 h (Figure 6a,b) and 48 h (Figure 6c,d) for pure PMMA and PMMA/Au samples. In the SEM images, it can be clearly seen that *C. albicans* is exclusively present in the yeast form. No hyphae were formed even after 48 h of incubation. We have defined the extent of yeast adhesion as the ratio between the number of cells and the sample area, and, therefore, it is expressed in μm*^−^*^2^. After 24 h, the extent of yeast adhesion for the unmodified PMMA was 0.00157 μm*^−^*^2^, while it was 0.00054 μm*^−^*^2^ for PMMA/Au sample (Table 3). The adhesion after 48 h markedly increased; the extent of adhesion for unmodified PMMA was 0.00398 μm*^−^*^2^, and for PMMA/Au sample it was 0.00227 μm*^−^*^2^ (Table 3).

### 3.8. EDS Analysis

**Figure 7:**
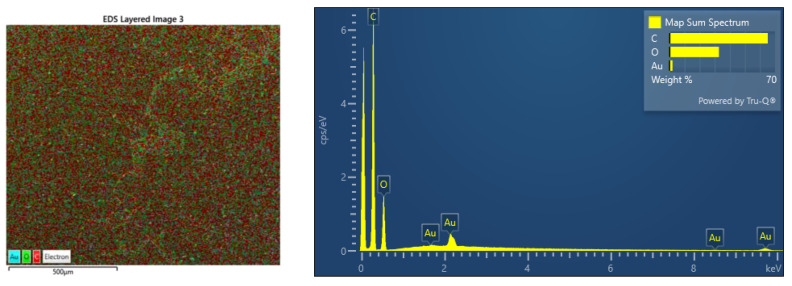
The presence of elements in the PMMA/Au samples by EDS analysis. The left figure shows the distribution of the following elements: blue-Au, green-O, and red-C. The right figure shows the energy distribution of present elements.

EDS was performed on carbon-sputtered samples at a working distance of 10 mm with an open detection. In this way, all possible elements could be observed. EDS mapping was performed to check the distribution of elements in PMMA/Au. The EDS analysis (Figure 7) showed that Au NP were homogenously redistributed and no strong agglomeration was observed.

## 4. Discussion

The aim of this study was to investigate the effects of Au nanoparticles on the tensile strength, color properties, roughness, surface zeta potential, and contact angle of PMMA denture base materials, as well as their effect on the growth and adhesion extent of *C. albicans* on the material surface. 

The size of the synthesized NPs was determined by the procedure outlined by the Haiss procedure [53] from the surface plasmon resonance maximum at ~520 nm. The average particle size determined was 11 nm, while the colloid concentration was estimated to be 4.7 nM. Even though the miscibility of Au aqueous colloidal solution and methyl methacrylate monomer is not good, we presume that citrate molecules which were used as both stabilizing and reducing agents enhanced their miscibility to an extent due to the presence of various oxygen-containing groups on both the citrates and methyl methacrylate monomers. 

Based on the CIELAB parameters, the color properties of base PMMA material (L* 53.8, a* 21.1, b* 12.9) are very similar to gingiva [56] (L* 57.5, a* 22.2, b* 14.4). The addition of Au NPs to PMMA only slightly changes the CIELAB color parameters of the base material, as evidenced from Table 1 (L* 55.5, a* 19.6, b* 11.4). The total color difference (ΔE) parameter between these two samples was 2.7 which would make the color difference between these samples distinct, but acceptable [57].

Contact angle measurements qualitatively reveal whether surfaces are hydrophilic (<90°) or hydrophobic (>90°). Both base PMMA and Au-modified PMMA were hydrophilic, however, Au-modified PMMA had a lower contact angle than the base material (55.6° vs. 76.5°). It was previously reported that the addition of nanoparticles reduces the contact angle of PMMA-based materials, in the case of TiO_2_, Ag, and Au [48,58,59]. *C. albicans* can exist as either hydrophilic or hydrophobic cells depending on environmental and growth conditions. Growth temperature is one of the most important factors regarding the cell surface hydrophobicity; at growth temperatures of 23–30 °C, the cell walls of *C. albicans* have hydrophobic proteins exposed on the cell surface, while at 37 °C conventional hydrophobicity measurements indicate that the cells are hydrophilic [60]. In this case, because the growth temperature for this particular study was 37 °C, the yeast cells in our study can be considered hydrophilic.

The PMMA and Au-modified PMMA are both negatively charged above a pH value of ~4.3, which is the isoelectric point of the samples. However, the magnitude of the negative charge is larger for Au-modified PMMA at pH values close to neutral, therefore, it can be concluded that in the salivary pH regime, the Au-modified PMMA should have a more negative charge. Considering the fact that *C. albicans* and fungi in general are negatively charged [61,62], repulsive forces between the fungi and PMMA/Au should be larger than for the base material.

The surface roughness is markedly different between the base material and PMMA/Au, where the base PMMA has two times higher roughness than the PMMA/Au. Sawant et al. noticed the same effect when they compared unmodified and Ag-modified polycaprolactam surfaces, where the nanoparticle-modified surface had a smoother surface evidenced by AFM. On the other hand, some authors noted that the addition of low amounts of nanoparticles increase the surface roughness of PMMA-based materials [13], up to a certain point, but upon further increase, the surface roughness decreases. It is known that surface roughness is a paramount factor regarding the success rate of denture materials and that highly polished materials are less susceptible to microbial adhesion [63,64]. 

The tensile test results of PMMA and PMMA/Au showed that the tensile strength significantly decreases upon the addition of Au NPs, and Shore D hardness tests showed a slight increase in the hardness of the composite material compared to the base material. This is in line with the results obtained by Adamović et al. [11] who showed that flexural strength and elastic modulus of PMMA/Au composites decreased as the wt% of added Au NPs increased. The authors also noticed a slight increase in the Vickers microhardness upon the addition of Au NPs. The authors argued that the addition of NPs to the resin increased the amount of unreacted monomer which, in turn, led to worse mechanical properties. Furthermore, NP agglomerates can act as stress centers and can lead to the formation of cracks and pores in the material [13]. Elshereksi et al. [65] also noticed an increase in the hardness of PMMA when modified with barium titanate nanoparticles. This increase in hardness can be explained by the high hardness of the dispersed Au nanoparticles.

As can be seen from the results of yeast adhesion, *C. albicans* cells adhere in larger quantities to the surface of the base PMMA material when compared to the PMMA/Au material. The adhesion extent of *C. albicans* increased with incubation time for all the samples, however, it can be clearly seen from these values that modification of PMMA with Au decreases the extent of yeast cells adhesion by 66% after 24 h and by 43% after 48 h. The yeast adheres to the surfaces based on specific physico-chemical characteristics such as hydrophobicity, surface charge, and surface roughness. As mentioned earlier in the discussion, the yeast can be considered hydrophilic, which should make the PMMA/Au material a better surface for yeast growth, however, this is not the case. On the other hand, PMMA/Au has a slightly lower zeta potential than base PMMA, which enhances the repulsive electrostatic forces between yeast cells and the material surface. Furthermore, PMMA/Au has a much smoother surface, which reduces the adhesion extent of *C. albicans* in this work on the PMMA/Au material surface. The difference in magnitude of these three factors between the base PMMA and PMMA/Au material is the largest for the surface roughness, which makes it the prime factor influencing the extent of adhesion for these particular samples. However, some authors have also noted that gold has a fungicidal effect and it is highly likely that the improved antimicrobial properties of PMMA/Au can be ascribed, at least in part, to this fungicidal effect of Au NPs.

## 5. Conclusions

In this study, the effect of Au NPs on the properties of PMMA-based dental material was explored. The material’s CIELAB color properties, wettability, surface charge, surface roughness, tensile strength, and hardness, as well as the growth of *C. albicans* yeast during a period of 48 h were examined.

A few general conclusions can be made based on the insights gained from this study:

1. The addition of Au NPs decreases the tensile strength and slightly increases the hardness of the base PMMA material.
2. The addition of Au NPs does not significantly affect the color of the base material, but does affect its wettability, surface charge, and roughness.
3. The addition of Au NPs improves the resistance of the base material to the adhesion of *C. albicans* by 66% after 24 h and by 43% after 48 h.

This preliminary work has shown that a simple, yet cost-effective method of synthesizing Au NPs and the subsequent modification of PMMA-based dental material can enhance the durability of PMMA material in regard to the growth of *C. albicans* yeast and the possible formation of microbial biofilm, without severely impacting the mechanical properties necessary to withstand mastication and oral health care practices. Further studies need to be done to optimize the amount of added Au NPs and to investigate other approaches to synthesize Au NPs in solvents fully compatible with methyl methacrylate monomers.

## Figures and Tables

**Table 1:** CIE L*a*b* parameters of pure PMMA and PMMA enriched with Au NPs.

	L*	a*	b*
PMMA	53.82 ± 1.06	21.12 ± 1.09	12.93 ± 0.70
PMMA/Au	55.47 ± 1.46	19.59 ± 0.89	11.43 ± 0.41

**Table 2:** The mean values with standard deviations of the maximum tensile strength (σ_M_), corresponding strain (ε_M_) and Shore D hardness values obtained by the Shore method of PMMA and PMMA/Au samples.

	σ_M_/MPa	ε_M_/%	Shore D
PMMA	56.7 ± 9.5	3.9 ± 1.0	22.2 ± 0.6
PMMA/Au	34.7 ± 2.8	2.8 ± 0.3	22.7 ± 0.9

**Table 3:** The values of yeast cell adhesion extent defined as the number of cells divided by the sample area expressed in μm*^−^*^2^ for PMMA and PMMA/Au samples after 24 h and 48 h of incubation. The mean and standard deviation values were obtained from a total of 25 micrographs and a total surface area of 1,114,566.38 μm^2^.

	24 h	48 h
PMMA	0.00157 ± 0.00120	0.00398 ± 0.00129
PMMA/Au	0.00054 ± 0.00095	0.00227 ± 0.00226

## Data Availability

All data that support the findings of this study are included within the article.
